# Differences in Glucose Metabolism Between Single Memory Domain and Multidomain Subjective Cognitive Decline: A Longitudinal Study From SILCODE

**DOI:** 10.1111/cns.70264

**Published:** 2025-05-27

**Authors:** Min Wei, Luyao Wang, Xianfeng Yu, Wenjing Hu, Min Wang, Qi Zhang, Tengfei Guo, Jiayi Zhong, Chenyang Li, Jiehui Jiang, Ying Han

**Affiliations:** ^1^ Department of Neurology Xuanwu Hospital of Capital Medical University Beijing China; ^2^ Institute of Biomedical Engineering, School of Life Sciences Shanghai University Shanghai China; ^3^ Department of Neurology The First Affiliated Hospital of Anhui Medical University Hefei China; ^4^ Institute of Biomedical Engineering Shenzhen Bay Laboratory Shenzhen China; ^5^ State Key Laboratory of Digital Medical Engineering, Key Laboratory of Biomedical Engineering of Hainan Province, School of Biomedical Engineering Hainan University Sanya China; ^6^ National Clinical Research Center for Geriatric Diseases Beijing China; ^7^ The Central Hospital of Karamay Xinjiang China

**Keywords:** Alzheimer's disease, FDG‐PET, plasma AD biomarkers, subjective cognitive decline

## Abstract

**Background:**

Glucose metabolism and plasma biomarkers have emerged as important early markers in Alzheimer's disease. Different subtypes (single memory domain, multidomain) of subjective cognitive decline (SCD) may represent distinct stages of disease progression, but the differences in glucose metabolism remain unclear. This study focused on exploring the differences in glucose metabolism between different SCD subtypes and the correlation with plasma biomarkers based on ^18^F‐FDG PET.

**Methods:**

In this study, thirty‐three normal controls (NCs), thirty‐five individuals with single memory domain SCD (sd‐SCD), thirty‐nine individuals with multidomain SCD (md‐SCD), and twenty‐one cognitively impaired (CI) individuals were involved. We investigated the standardized uptake value ratio (SUVR) and voxel differences between the sd‐SCD and md‐SCD groups followed by FDR and GRF corrections, with an average follow‐up time of 44.98 ± 16.49 months. Correlation analyses were employed to assess relationships between FDG‐PET SUVR and neuropsychological scales as well as plasma biomarkers. Finally, Kaplan–Meier survival analysis was used to investigate the risk of cognitive decline conversion among SCD subgroups.

**Results:**

After controlling for the effects of covariates, the following brain regions showed voxel differences and lower SUVR in md‐SCD groups, including right anterior cingulate and paracingulate gyri (ACG.R, *p =* 0.003), left anterior cingulate and paracingulate gyri (ACG.L, *p =* 0.003), right middle temporal gyrus (MTG.R, *p =* 0.004), and right inferior temporal gyrus (ITG.R, *p =* 0.001), compared to the sd‐SCD group. SUVR of ACG.R was correlated with plasma Aβ42/40 (*r* = 0.435, *p* = 0.006) and AVLT‐N7 score (*r* = 0.347, *p* = 0.031) in the md‐SCD group while none of the correlations existed in the sd‐SCD group. SUVR of MTG.R was also correlated with the AVLT‐N7 score (*r* = 0.246, *p* = 0.035) across SCD individuals. The SCD individuals with positive plasma Aβ42/40, p‐tau181, and glucose metabolism in above four regions, or those in the md‐SCD group showed an elevated risk of cognitive conversion in comparison to the controls.

**Conclusions:**

Differences in glucose metabolism could be observed between the md‐SCD and sd‐SCD groups. SCD participants in the md‐SCD group, or those with positive biomarkers, might represent a higher risk of cognitive decline conversion.

## Graphical abstract

The study highlights differences in glucose metabolism between two SCD subtypes, indicating that md‐SCD individuals or SCD individuals with high‐risk factors including positive plasma Aβ42/40, p‐tau181, and glucose metabolism biomarkers, might exhibit an increased risk of cognitive decline. This provides a new valuable clinical biomarker for timely diagnosis, identifying high‐risk individuals, and predicting cognitive conversion.

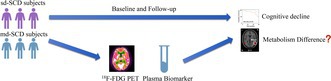

## 1 Introduction

Alzheimer's disease (AD) is a neurological disease which has a high disease burden and an extremely increasing prevalence [1, 2, 3, 4]. Due to the lack of clinical treatments for AD, early detection of AD is particularly important [5]. Subjective cognitive decline (SCD) is marked by persistent self‐perceived decline in cognitive functioning, but objective cognitive assessment is not impaired [6]. People with SCD face an increased risk of developing cognitive abnormalities in contrast to normal [7]. However, given the heterogeneity in the etiology of SCD, differentiating its subtypes and identifying individuals at high risk for SCD becomes a critical endeavor.

The SCD‐Interview (SCD‐I), provided by the German Center for Neurodegenerative Diseases, assesses SCD across five cognitive domains. Among SCD individuals, only those reporting SCD in the memory domain are categorized into the single memory domain SCD (sd‐SCD) group, while those reporting SCD in one or more cognitive domains apart from memory are categorized as the multidomain SCD (md‐SCD) group [8, 9]. Previous research suggested that individuals classified under md‐SCD exhibited a higher degree of global amyloid accumulation compared to those classified under sd‐SCD, which indicated that sd‐SCD and md‐SCD might be potential subtypes for SCD [8]. However, more evidence from biomarkers is required to define the differences between sd‐SCD and md‐SCD.

Dysfunctional brain glucose metabolism is tightly linked to the neuropathology of AD, and the compromised glucose metabolism might trigger tau phosphorylation, amyloid precursor protein process changes, and amyloidogenic deposition [10, 11, 12]. The previous studies proposed that hypometabolism based on ^18^F‐fluorodeoxyglucose positron emission tomography (^18^F‐FDG PET) imaging could be a potential biomarker for SCD [13, 14, 15]. However, it is still unknown whether glucose metabolism shows differences in sd‐SCD and md‐SCD. Moreover, plasma biomarkers, such as amyloid‐β42/40 (Aβ42/40), neurofilament light (NfL), phosphorylated tau181 (p‐tau181), and glial fibrillary acidic protein (GFAP), have been widely used in AD and SCD studies [16, 17, 18, 19, 20]. Reduced plasma Aβ42/40 level is increasingly recognized as an alternative marker for detecting amyloid accumulation and identifying individuals with high‐risk evolvement [21]. Plasma p‐tau181, as a phosphorylated tau protein, was correlated with longitudinal clinical progression, recognizing positive Aβ‐PET and predicting positive tau‐PET in the elderly [22, 23]. Plasma NfL serves as a signal of neuroaxonal injury and elevates across the severity spectrum of AD [24, 25]. Enhanced plasma GFAP concentrations might refer to reactive astrogliosis and perform excellently in tracking AD neuropathology [26, 27]. Given the essential effects of glucose metabolism and plasma biomarkers in the early phases of AD, it is also required to investigate the correlations between glucose metabolism profiles and plasma biomarkers across SCD subtypes.

Therefore, this study aimed to investigate the differences in glucose metabolism between sd‐SCD and md‐SCD and whether these differences can be used as a new biomarker for predicting SCD conversion. In addition, this study also aimed to compare the outcome risk of the two and the possible mechanisms responsible for their differences.

## 2 Methods

### 2.1 Participants

The study is a section of the Sino Longitudinal Study on Cognitive Decline (SILCODE), a registered ongoing exploration among the Han ethnic population in China (NCT03370744). Approval for this study was granted by the ethics committee at Xuanwu Hospital of Capital Medical University (2017 [046]), and the protocol is available on ClinicalTrials.gov. Our study obtains written informed consent from every subject or their caregivers. Thirty‐three normal control (NC) individuals, seventy‐four SCD individuals (thirty‐five sd‐SCD, thirty‐nine md‐SCD), and twenty‐one cognitively impaired (CI) individuals were covered in our study between May 2018 and October 2022. All subjects were right‐handed and aged sixty years or older. The NC group had cognitive test results inside the normal extent and had no sustained self‐perceived cognitive decline.

The inclusion criteria of SCD were based on the concept introduced by Jessen [6] and previous work [8]. These criteria encompassed: (1) self‐reported ongoing memory decline; (2) objective normal neuropsychological indicators; (3) failure to fulfill the criteria for mild cognitive impairment (MCI) [28] or dementia [29]. Exclusion criteria encompassed subjects with conditions like stroke, significant vascular lesions, severe anxiety, severe depression, psychiatric origin SCD, abnormal thyroid function, syphilis, anemia, traumatic brain injury, and so on [8]. The CI group comprised participants diagnosed with MCI and dementia, with MCI diagnosis relying on neuropsychological assessment [28] and AD dementia diagnosed in accordance with the NIA‐AA [29, 30]. All subjects in both the sd‐SCD and md‐SCD groups were longitudinally followed up, with an average follow‐up time of 44.98 ± 16.49 months. Conversion to cognitive decline status was considered if, compared to the baseline, the number of impaired cognitive domains (memory, language, and executive function) at follow‐up had increased by at least one or already met the diagnostic criteria for MCI [28].

### 2.2 Neuropsychological Assessment

The evaluation of subjective cognition was conducted using the Chinese version SCD‐I. The SCD‐I is a semi‐structured interview projected from a neurodegenerative center in Germany [31, 32, 33]. It evaluates SCD across different cognitive domains, involving memory, language, plan, attention, and others. Professional research doctors administer the assessments, probing subjects about specific alterations in five cognitive domains in recent years, as well as the specifics and timing of symptom onset (details are provided in Supporting Information).

All individuals were administered the Mini‐Mental State Examination (MMSE); memory function: Auditory Verbal Learning Test long‐term delayed recall (AVLT‐N5) and recognition (AVLT‐N7); executive function: Shape Trail Test A (STT‐A) and Shape Trail Test B (STT‐B); language function: Verbal Fluency Test (VFT) and Boston Naming Test (BNT), to assess cognition.

### 2.3 Imaging Acquisition

All FDG‐PET and MRI images were scanned with a 3.0T PET/MR scanner (Signa, GE Healthcare, USA) at Xuanwu Hospital. Prior to FDG‐PET, participants underwent a fasting period of more than six hours, and if their blood glucose was within 120 mg/dL, ^18^F‐FDG (3.7 MBq/kg) was intravenously injected for forty minutes before image acquisition. The FDG‐PET data were recorded using a time‐of‐flight ordered subset expectation maximization algorithm (matrix = 192 × 192, FOV = 350 × 350 mm^2^, slice number = 89, slice thickness = 2.78 mm, voxel size = 1.82 × 1.82 × 2.78 mm^3^). T1‐weighted images were performed using a magnetization‐prepared rapid gradient echo sequence (matrix = 256 × 256, FOV = 256 × 256 mm^2^, TR = 6.9 ms, TE = 2.98 ms, TI = 450 ms, FA = 12°, slice thickness = 1 mm, voxel size = 1 × 1 × 1 mm^3^).

### 2.4 Plasma Biomarker Extraction

After an overnight fast, venous blood was collected from participant in the morning via EDTA tubes. After centrifugation at 4°C with 3000 × g, the supernatant was retrieved as plasma and stored at −80°C for further analysis. Concentrations of plasma Aβ40, Aβ42, GFAP, p‐tau181, and NfL were measured by the Single Molecule Array (Simoa) HD‐X analyzer platform from Quanterix Corporation. P‐tau181 concentration was assessed using the Simoa pTau‐181 Advantage V2 Kit (Cat # 103714), while Aβ40, Aβ42, NfL, and GFAP concentrations were measured using the Simoa Neurology 4‐Plex E (N4PE) Advantage Kit (Cat # 103670) assay from Quanterix. All assays were repeated, and the average values were reported. Intra‐assay coefficients of variation (CV) for controls ranged from 1% to 8% for GFAP, 1% to 5% for Aβ40, 2% to 12% for NfL, 2% to 13% for Aβ42, and 1% to 10% for p‐tau181. The lower limits of detection of the GFAP, p‐tau181, Aβ40, Aβ42, and NfL assays were 0.441, 0.028, 0.384, 0.136, and 0.090 pg/mL, meanwhile, the lower levels of quantification were 2.890, 0.338, 1.020, 0.378, and 0.400 pg/mL.

### 2.5 Image Preprocessing

Firstly, the DICOM files of PET and T1‐weighted images were converted to NIfTI files by DCM2NII (https://people.cas.sc.edu/rorden/mricron/dcm2nii.html). Subsequently, segmentation of gray matter (GM), white matter (WM), and cerebrospinal fluid (CSF) from T1‐weighted images was performed using the CAT12 toolbox (http://dbm.neuro.uni‐jena.de/cat/). Next, PET images were coregistered to T1‐weighted images and normalized to the MNI standard space. To increase the signal‐to‐noise ratios, the images were smoothed with an 8 mm full width at half maximum Gaussian kernel. In addition, the PET images were normalized to the reference brain region (the whole brain was used as the reference region in this work) to calculate the standardized uptake value ratio (SUVR).

### 2.6 FDG‐PET Analysis

First, a voxel‐wise two‐sample t‐test was executed between sd‐SCD and md‐SCD group utilizing DPARSF version 5.2 (http://rfmri.org/DPARSF). Covariates such as age, sex, education, APOE, and plasma Aβ42/40 levels were regressed in all anaylses. Differences between the sd‐SCD and md‐SCD groups were observed using Gaussian random field (GRF) correction (at the voxel level *p* < 0.01, at the cluster level *p* < 0.05, voxels > 1000). Subsequently, we calculated SUVR values utilizing the Anatomical Automatic Labeling (AAL) template [34]. Then we compared the SUVR values of each region of interest (ROI) between the sd‐SCD and md‐SCD groups with a two‐sample t‐test, a Mann–Whitney U test and false discovery rate (FDR) correction (MATLAB R2020b, MathWorks Inc), where significance was set at *p* < 0.01 [35]. Brain regions were selected with voxel‐level and ROI‐level differences, followed by evaluating the SUVR variations in the AD continuum with one‐way analysis of variance (ANOVA), Kruskal–Wallis test, and post hoc test.

### 2.7 Correlation Analysis

To investigate the relationship between SUVR values and neuropsychological scales as well as plasma biomarkers, we performed partial correlation analysis. Sex, age, education, APOE, and plasma Aβ42/40 levels were regressed as covariates. Additionally, to explore whether there were different correlations, the analyses were performed on sd‐SCD group, md‐SCD group and all SCD separately. *p* < 0.05 was considered significant on statistics.

### 2.8 Kaplan–Meier Survival Analysis

In the SCD group, Kaplan–Meier survival analysis was performed between the sd‐SCD and md‐SCD groups. Furthermore, we divided all SCD subjects into high‐ risk and low‐risk groups based on SUVR and plasma biomarkers, and performed Kaplan–Meier survival analysis between these two groups. *p* < 0.05 was considered significant on statistics. The median SUVR value was used as the cutoff for each group, while the thresholds for plasma biomarkers in the SILCODE cohort were determined by Shenzhen Bay Laboratory (longitudinal cohort Greater‐Bay‐Area Healthy Aging Brain Study, China). The thresholds of plasma Aβ42/40 ratio and p‐tau181 divided the SCD individuals into different plasma staging profiles: A−/A+ and T−/T+ [36]. The defined conversion to cognitive decline was utilized as the endpoint event for conversion and assessed for survival through the log‐rank test.

### 2.9 Statistical Analysis

We used the Anderson–Darling test to assess the normal distribution of continuous variables. Chi‐square tests, two‐sample t‐tests, and Mann–Whitney *U* tests were performed for group comparisons on demographic informatics.

## 3 Results

### 3.1 Participants Assessment

The demographic and clinical characteristics of participants are listed in Table 1. There were no significant differences in age, sex, years of education, and *APOE* between sd‐SCD and md‐SCD groups, as well as between NC and SCD groups. The sd‐SCD and md‐SCD groups did not show a statistical difference between groups on neuropsychological scales as well as the NC and SCD groups (*p* > 0.05). Plasma Aβ42/40 did not exhibit a significant difference between the NC and sd‐SCD groups. While the sd‐SCD and md‐SCD groups did not show significance regarding the plasma biomarkers p‐tau181, NfL, and GFAP, they did show significance for Aβ42/40 (*p* < 0.05). In comparison to the SCD group, the CI group was older (*p* < 0.05), and there were statistically significant differences observed in terms of the number of APOE ε4 carriers, plasma biomarkers, and neuropsychological scale results (*p* < 0.05). Among the 74 subjects with SCD (sd‐SCD+md‐SCD), all showed memory loss, with 33.78% also showing decreased language function and 16.22% showing decreased planning ability. Within the 39 subjects comprising the md‐SCD group, 64.10% showed decreased language function, 30.77% showed decreased planning ability, and 56.41% showed decreased attention function.

**TABLE 1:** General characteristics of the participants at baseline.

	Baseline				Baseline *p* value				
	NC (*n* = 33)	sd‐SCD (*n* = 35)	md‐SCD (*n* = 39)	CI (*n* = 21)	NC versus SCD	NC versus sd‐SCD	sd‐SCD versus md‐SCD	md‐SCD versus CI	SCD versus CI
Sex (M, *n*%)	8 (24.24%)	11 (31.43%)	13 (33.33%)	6 (28.57%)	0.393^a^	0.509^a^	0.861^a^	0.705^a^	0.737^a^
Age (year)	65.303 ± 3.477	65.257 ± 4.182	67.359 ± 5.065	72.048 ± 6.459	0.558^c^	0.626^c^	0.109^c^	0.005^c^	<0.001^c^
Education	12.455 ± 3.042	13.229 ± 2.636	12.795 ± 2.858	12.857 ± 2.505	0.301^c^	0.218^c^	0.499^c^	0.795^c^	0.986^c^
APOEε4 (carrier, *n*, %)	5 (15.15%)	7 (20.00%)	16 (41.03%)	15 (71.43%)	0.600^a^	0.083^a^	0.051^a^	0.025^a^	0.001^a^
Memory^d^ (Yes; *n*, %)	/	35 (100.00%)	39 (100.00%)	/	/	/	/	/	/
Language^d^ (Yes; *n*, %)	/	/	25 (64.10%)	/	/	/	/	/	/
Plan^d^ (Yes; *n*, %)	/	/	12 (30.77%)	/	/	/	/	/	/
Attention^d^ (Yes; *n*, %)	/	/	22 (56.41%)	/	/	/	/	/	/
Others^d^ (Yes; *n*, %)	/	/	5 (12.82%)	/	/	/	/	/	/
Plasma Aβ42/40	0.062 ± 0.012	0.061 ± 0.010	0.054 ± 0.014	0.052 ± 0.011	0.034^c^	0.535^b^	0.006^c^	0.321^c^	0.029^c^
Plasma p‐tau181	1.796 ± 0.718	1.664 ± 0.517	2.194 ± 1.064	4.025 ± 2.103	0.494^c^	0.740^c^	0.079^c^	<0.001^c^	<0.001^c^
Plasma NfL	17.516 ± 9.244	15.468 ± 9.948	22.101 ± 13.965	26.777 ± 11.852	0.606^c^	0.641^c^	0.060^c^	0.019^c^	<0.001^c^
Plasma GFAP	96.569 ± 53.283	87.660 ± 26.529	111.180 ± 60.450	233.121 ± 96.726	0.625^c^	0.990^c^	0.299^c^	<0.001^c^	<0.001^c^
AVLT‐N5	7.636 ± 1.901	7.686 ± 2.097	7.308 ± 2.079	1.684 ± 2.212	0.848^c^	0.825^b^	0.278^c^	<0.001^c^	<0.001^c^
AVLT‐N7	22.667 ± 1.472	22.543 ± 1.400	22.103 ± 1.903	16.684 ± 4.230	0.320^c^	0.611^c^	0.433^c^	<0.001^c^	<0.001^c^
VFT	20.424 ± 4.542	19.600 ± 3.423	18.769 ± 3.808	12.333 ± 5.257	0.131^b^	0.423^b^	0.143^c^	<0.001^c^	<0.001^c^
BNT	25.212 ± 2.484	24.886 ± 2.665	24.974 ± 3.133	19.524 ± 4.854	0.916^c^	0.892^c^	0.900^c^	<0.001^b^	<0.001^c^
STT‐A	55.281 ± 14.445	55.543 ± 18.038	59.795 ± 17.321	113.700 ± 54.322	0.554^c^	0.930^c^	0.298^c^	<0.001^c^	<0.001^c^
STT‐B	130.500 ± 32.617	132.657 ± 29.125	142.897 ± 32.804	250.813 ± 108.247	0.613^c^	0.868^b^	0.225^c^	<0.001^c^	<0.001^c^
MMSE	29.030 ± 1.159	28.853 ± 1.048	28.703 ± 1.631	21.381 ± 5.878	0.302^c^	0.322^c^	0.804^c^	<0.001^c^	<0.001^c^
HAMA	4.273 ± 3.125	3.800 ± 2.837	4.615 ± 3.491	5.667 ± 5.102	0.807^c^	0.562^c^	0.360^c^	0.662^c^	0.421^c^
HAMD	3.879 ± 4.314	2.457 ± 2.873	3.821 ± 3.268	5.333 ± 5.295	0.643^c^	0.145^c^	0.052^c^	0.460^c^	0.092^c^
GDS	2.455 ± 2.265	1.914 ± 1.292	2.436 ± 2.023	2.737 ± 1.910	0.895^c^	0.619^c^	0.400^c^	0.373^c^	0.152^c^
Plasma Aβ42/40 (positive, *n*, %)	14 (42.42%)	15 (42.86%)	30 (76.92%)	16 (76.19%)	0.631^a^	0.971^a^	0.003^a^	0.949^a^	0.001^a^
Plasma p‐tau181 (positive, *n*, %)	4 (12.12%)	4 (11.43%)	13 (33.33%)	17 (80.95%)	0.389^a^	0.929^a^	0.025^a^	<0.001^a^	<0.001^a^

*Note:* *p* value: significant differences.

^a^ Chi‐square test.

^b^ *t*‐test.

^c^ Mann–Whitney *U* test.

^d^ SCD‐I domains (decline in memory, language, plan, attention, and others).

### 3.2 Voxel and SUVR Analysis

**FIGURE 1:**
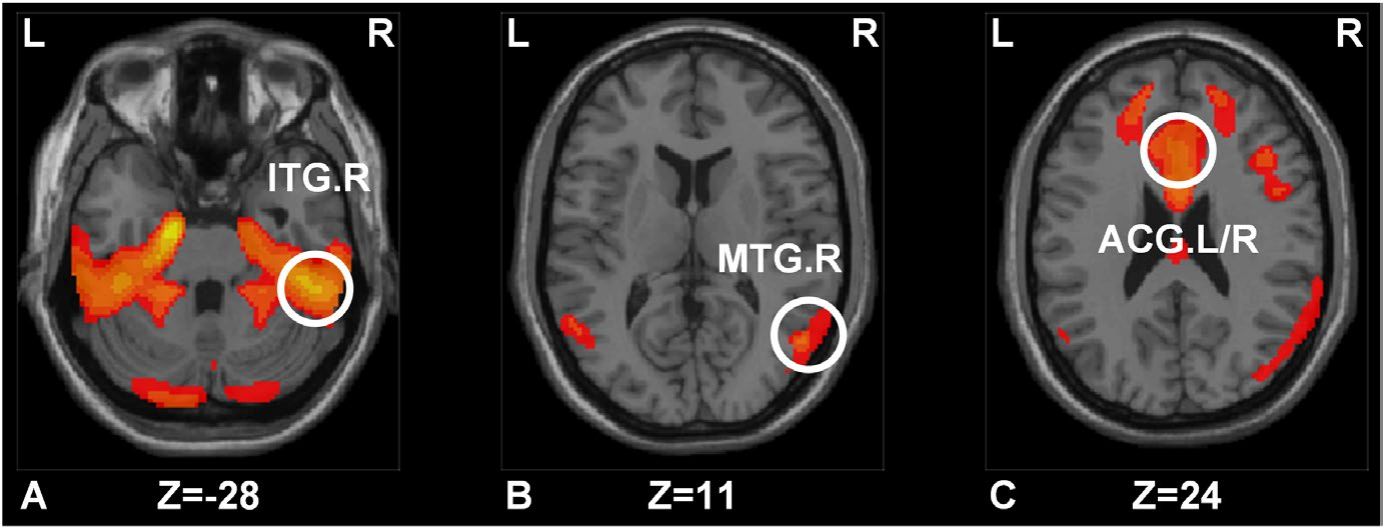
Comparison of voxel levels between sd‐SCD and md‐SCD groups in four brain regions (A–C). Differences between sd‐SCD and md‐SCD groups at the voxel level (regions where the difference in SUVR between the two groups and the AD continuum are simultaneously significant and meaningful). ACG.L, left anterior cingulate and paracingulate gyri; ACG.R, right anterior cingulate and paracingulate gyri; ITG.R, right inferior temporal gyrus; MTG.R, right middle temporal gyrus.

**FIGURE 2:**
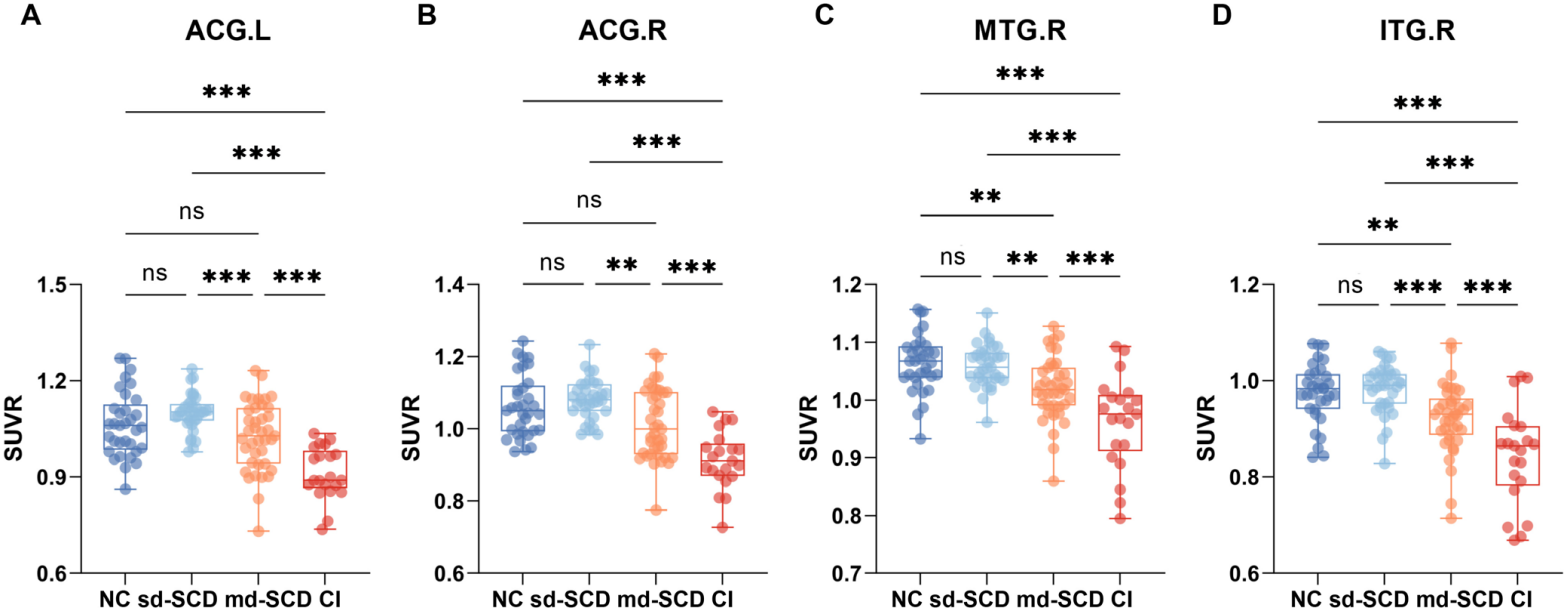
Metabolism in four statistically significant regions along the cognitive continuum. The A–D plots show the SUVR of the four brain regions. The SUVR values of the four brain regions, ACG.L, ACG.R, MTG.R, and ITG.R, were significantly different (*p* < 0.05) between the sd‐SCD and md‐SCD groups and between the md‐SCD and CI groups. ns, not significant, *p < 0.05, **p < 0.01, ***p < 0.001. ACG.L, left anterior cingulate and paracingulate gyri; ACG.R, right anterior cingulate and paracingulate gyri; CI, cognitively impaired; ITG.R, right inferior temporal gyrus; md‐SCD, multidomain SCD; MTG.R, right middle temporal gyrus; NC, normal control; sd‐SCD, single memory domain SCD; SUVR, standardized uptake value ratio.

The voxel analysis results revealed differences in multiple brain regions between sd‐SCD and md‐SCD groups. At the same time, we calculated the difference in SUVR values between these groups and those in the AD continuum. Notably, the brain regions that exhibited differences both in voxel size level and ROI level included the right middle temporal gyrus (MTG.R, *p* = 0.004), the right inferior temporal gyrus (ITG.R, *p* = 0.001), the left anterior cingulate and paracingulate gyri (ACG.L, *p* = 0.003), and the right anterior cingulate and paracingulate gyri (ACG.R, *p* = 0.003) (Figure 1 and Table 2), with these brain regions showing lower SUVR in md‐SCD groups compared to sd‐SCD groups. Figure 2A–D shows the results of the comparison of SUVR among the NC, sd‐SCD, md‐SCD, and CI groups. Statistically significant differences were observed among four groups for each brain region (Figure 2A, *p* < 0.001, *F*(3,124) = 20.24; Figure 2B, *p* < 0.001, *H* = 21.32; Figure 2C, *p* < 0.001, *F*(3,124) = 18.83; Figure 2D, *p* < 0.001, *F*(3,124) = 20.76). Post hoc analyses showed significant metabolic differences between the sd‐SCD and md‐SCD groups (ACG.L, Figure 2A, *p* < 0.001; ACG.R, Figure 2B, *p* = 0.003; MTG.R, Figure 2C, *p* = 0.003; ITG.R, Figure 2D, *p* < 0.001), as well as between the md‐SCD and CI groups (ACG.L, Figure 2A, *p* < 0.001; ACG.R, Figure 2B, *p* < 0.001; MTG.R, Figure 2C, *p* < 0.001; ITG.R, Figure 2D, *p* < 0.001).

**TABLE 2:** Brain regions with significant differences between sd‐SCD and md‐SCD based on voxel level and ROI level.

Voxels (Cluster size)	*X*	*Y*	*Z*	Intensity	Region
1948	52	−40	−28	6.282	ITG.R
1208	55	−65	11	3.646	MTG.R
1454	−2	21	24	4.403	ACG.L/R

Abbreviations: ACG.L/R, left/right anterior cingulate and paracingulate gyri; ITG.R, right inferior temporal gyrus; MTG.R, right middle temporal gyrus; *X*, *Y*, *Z*, Montreal Neurological Institute.

### 3.3 Correlation Analysis

**FIGURE 3:**
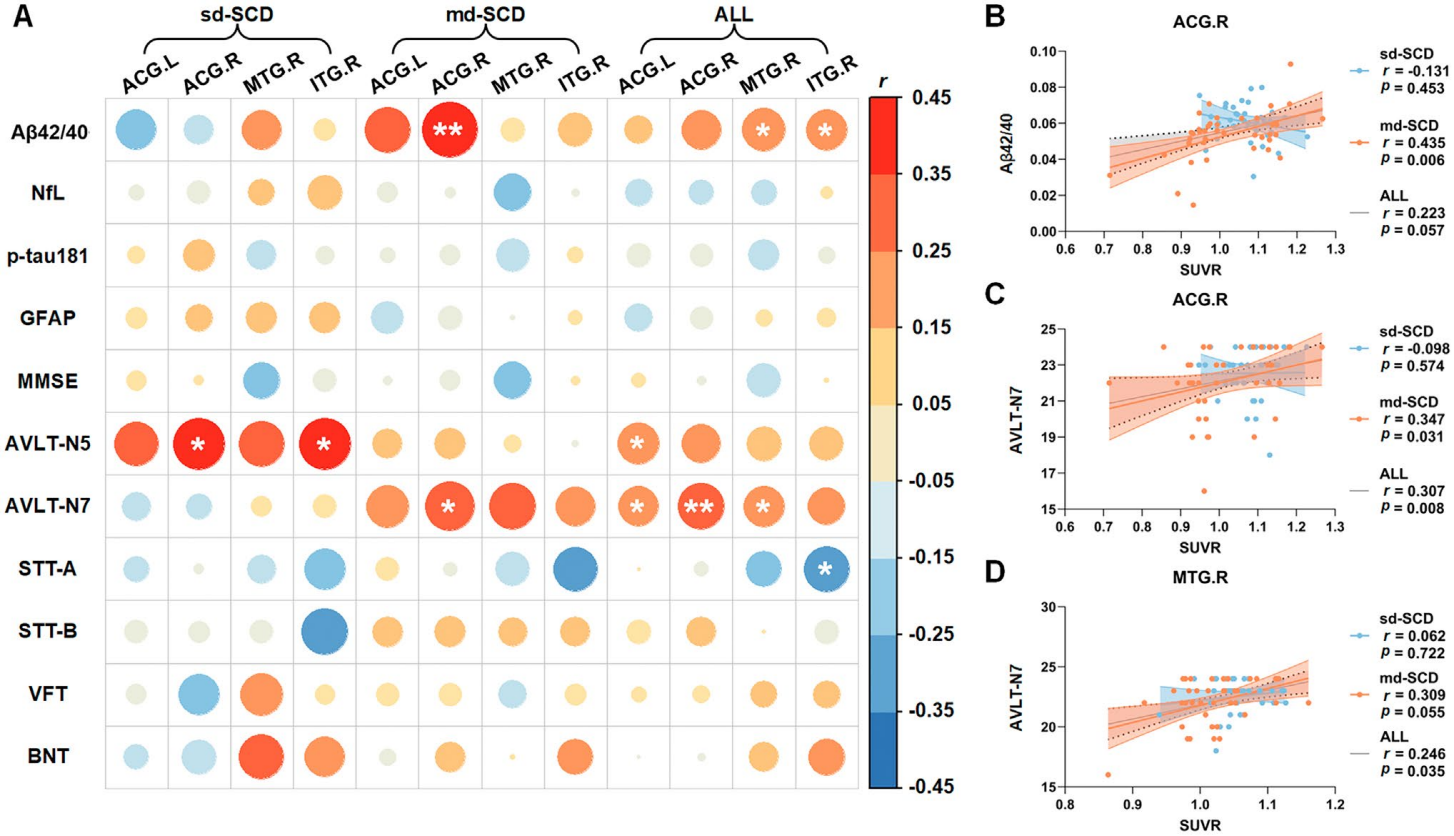
The correlation among four regions SUVR, plasma biomarkers, and neuropsychological scale scores (A). Elevated glucose metabolism in ACG.R was associated with elevated plasma Aβ42/40 (B) and elevated AVLT‐N7 (C). Reduced glucose metabolism in MTG.R metabolism was associated with decreased AVLT‐N7 (D). *p < 0.05, **p < 0.01. Aβ42/40, amyloid‐β42/40 ratio; ACG.R, right anterior cingulate and paracingulate gyri; AVLT‐N7, Auditory Verbal Learning Test recognition; SUVR, standardized uptake value ratio.

Figure 3A presents the correlation between SUVR and plasma biomarkers, as well as neuropsychological scale scores. In Figure 3B–D, it is observed that the metabolism of ACG.R was positively correlated with plasma Aβ42/40 (*r* = 0.435, *p* = 0.006) and the AVLT‐N7 score in the md‐SCD group. Notably, these correlations remained significant or marginal when the sd‐SCD and md‐SCD groups were combined into the SCD group. Similarly, MTG.R metabolism was positively correlated with the AVLT‐N7 score across SCD individuals, although this correlation was not presented in the sd‐SCD group. Within the SCD group, ACG.R metabolism exhibited a positive correlation with the AVLT‐N7 score (*r* = 0.307, *p* = 0.008), as well as MTG.R metabolism demonstrated a positive correlation with the AVLT‐N7 score (*r* = 0.246, *p* = 0.035). These findings suggest that reduced ACG.R metabolism is associated with decreased plasma Aβ42/40 and decreased recognition test scores, while reduced MTG.R metabolism is associated with decreased AVLT‐N7 scores.

### 3.4 Survival Analysis

**FIGURE 4:**
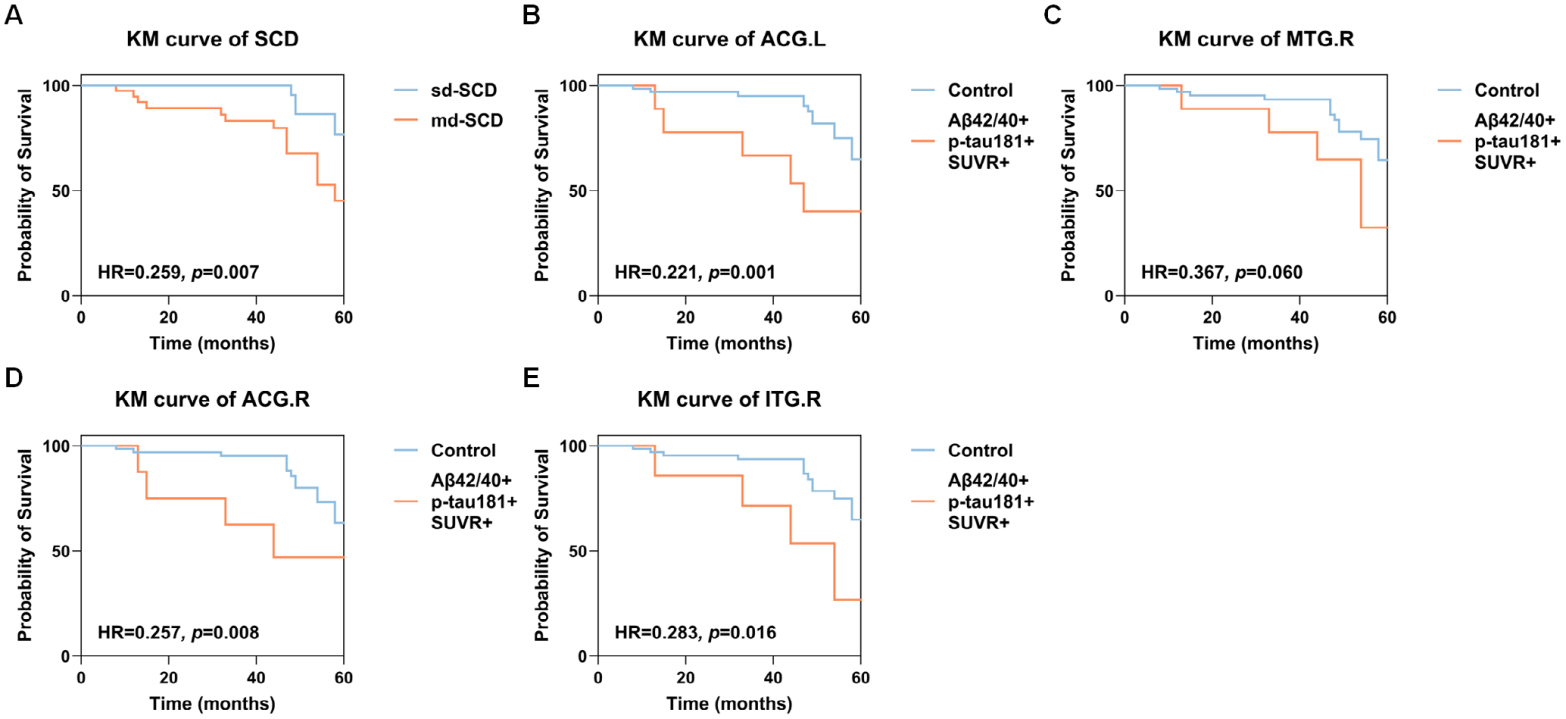
Kaplan–Meier survival curves based on SUVR combining the four brain regions of ACG.L, MTG.R, ACG.R, and ITG.R, respectively, plasma Aβ42/40 and p‐tau181. Combining the SUVR values of each of the four regions (A–E) with plasma Aβ42/40 and p‐tau181 allowed for better identification of conversion risk. Aβ, amyloid‐β; p‐tau181, phosphorylated tau181; SUVR, standardized uptake value ratio; Control, Each group of non‐simultaneous positive subjects; HR, hazard ratio.

In survival analysis, the md‐SCD group showed an elevated risk of cognitive decline conversion in comparison to the sd‐SCD group (*p* = 0.007, Figure 4A). Figure 4B shows that there is a statistically significant disparity between the subjects in the SCD group who also fulfilled the SUVR positivity, Aβ42/40 positivity, and p‐tau181 positivity of ACG.L, in contrast to the control group (*p* = 0.001). Similarly, in Figure 4C, SCD subjects who met MTG.R for SUVR positivity, Aβ42/40 positivity, and p‐tau181 positivity differed marginally from the control group (*p* = 0.060). Figure 4D highlights a noteworthy difference between SCD subjects meeting the criteria for ACG.R SUVR positivity, Aβ42/40 positivity, and p‐tau181 positivity compared to controls (*p* = 0.008). SCD subjects who met the criteria for ITG.R for SUVR positivity, Aβ42/40 positivity, and p‐tau181 positivity were found to be significantly different from the control group (*p* = 0.016) in Figure 4E. It is evident that the SUVR values of each of the four regions, in combination with plasma Aβ42/40 and p‐tau181, demonstrate excellent competence in distinguishing between high‐ and low‐risk groups.

## 4 Discussion

The objective of the study is to explore the differences in glucose metabolism between sd‐SCD and md‐SCD, whether this difference can be used as a new biomarker for predicting SCD conversion, and to compare the outcome risk of the two and the possible mechanisms responsible for their differences. This study indicates md‐SCD participants exhibited lower glucose metabolism compared to those with sd‐SCD. As we know, this is one of the pioneer studies on glucose metabolism among SCD subtypes. Moreover, we included baseline and longitudinal follow‐up data simultaneously in longitudinal survival analysis; these results also demonstrate that md‐SCD participants or SCD participants with high‐risk factors including positive plasma Aβ42/40, p‐tau181, and glucose metabolism biomarkers in four regions, exhibit a higher risk of cognitive decline, revealing substantial predictive value.

Our results indicate that md‐SCD subjects exhibited lower glucose metabolism in MTG.R, ITG.R, ACG.L, and ACG.R regions compared to sd‐SCD. Previous studies reported that in AD, the regions affected by the characteristic pattern of hypometabolism in the brain include the posterior cingulate and parietotemporal association cortices [37, 38, 39]. In previous research, hypometabolism has been observed in ACG and temporal‐associated cortices in SCD [40]; moreover, recent studies have highlighted the significance of reduced metabolism in the MTG.R and ITG as a characteristic feature of SCD [14], which is consistent with our findings. But with some new findings, excitingly, the differential metabolic patterns could be observed in ACG.R, ACG.L, MTG.R, and ITG.R between sd‐SCD and md‐SCD in our study, which was not reported in previous studies. At both voxel and ROI levels, the differences in glucose metabolism identified among subgroups of SCD are in line with alterations observed in the AD continuum. This supports the notion that reduced glucose metabolism is predictive of cognitive decline as a biomarker associated with AD progression and further substantiates the necessity for stratification within the SCD status longitudinally [41, 42, 43]. Our results observed no obvious difference in SUVR between the NC and sd‐SCD groups, indicating that sd‐SCD, as an early stage of SCD, might closely resemble NC state to some extent. Prominent cognitive complaints may imply a relatively early period in the dementia continuum, such as pre‐MCI, further emphasizing the role of stratification during the SCD stage [44]. For future applications of the differences in glucose metabolism, it could be developed as a personalized biomarker for predicting cognitive conversion. Overall, the glucose metabolism of SCD subtypes may be a valuable biomarker for SCD exploration in Chinese, which could contribute to enriching the disease spectrum of AD.

The survival curve results in this study revealed that the md‐SCD group had a higher risk of conversion than sd‐SCD, suggesting that the classification of SCD subgroups based on multiple domains has the potential to recognize the risk of cognitive impairment conversion. Following past studies, polymeric scores for SCD domains could serve as optimal predictors for AD pathology, which is in line with our result to some extent [31]. Similar findings have been observed in studies of MCI, where cortical thickness undergoes progressive atrophy as individuals transition from single‐domain MCI to multidomain MCI, representing phases in MCI progression [45]. As a primary synaptic biomarker of AD [46], the hypometabolism FDG‐PET is linked to cognitive decline and conversion to dementia [47]. This further elucidates the clinical significance of stratifying SCD, indicating that increased damage in SCD‐I may augment AD pathology deposition, consequently resulting in a greater risk of conversion of cognitive decline. Although the capability of biomarkers has been mentioned in previous studies, it was without the stratification of SCD subgroups [48, 49, 50, 51, 52]. Previous research has identified the significant role of glucose metabolism in predicting cognitive progression in AD [53]. Moreover, studies have reported plasma biomarkers such as plasma Aβ42 and p‐tau181 might reflect changes in CSF and help predict AD before clinical onset [54]. And longitudinal studies have observed that a combination of biomarkers, such as plasma Aβ42/40, p‐tau181, and digital cognitive test, can predict Aβ‐PET positivity and disease progress [55]. Particularly, our results demonstrated that among all individuals with SCD, who exhibited glucose metabolism SUVR+ in ROI regions, along with plasma Aβ42/40+ and p‐tau181+, were found to be at a higher risk of cognitive conversion compared to the control group (Figure 4). This confirms the predictive value of the biomarker in the course of AD to a certain extent [56]. These results also highlight the significance of integrating plasma biomarkers and glucose metabolism to predict the conversion process by the ATN framework [30, 49, 57]. Given these discoveries, the potential mechanism that drives the conversion of cognitive function in SCD subtypes, as outlined in this study, could be beneficial for future research and clinical diagnosis. From another perspective, it is illustrated that SUVR and plasma biomarkers in ROI regions can recognize parts of SCD individuals that are susceptible to conversion, suggesting a strong capacity to differentiate between high‐ and low‐risk SCD and predict cognitive decline. The results demonstrated that our SCD subtypes for predicting survival and conversion were more meticulous and practical than the traditional single SCD states.

In addition, our findings revealed a consistent correlation between glucose metabolism in both ACG.R and MTG.R with AVLT‐recognition memory function across all subjects with SCD, which is similar to previous correlation results [58, 59]. Studies have reported that MTG is associated with episodic memory, possibly attributed to the decreased regional homogeneity and gray matter volume in the dorsal attention network among SCD participants [60]. The ACG might crucially modulate neuronal circuit plasticity involved in memory function by operating the hippocampus and subventricular zone [61]. Meanwhile, we observed that reduced glucose metabolism in the ACG.R correlated with plasma Aβ42/40 in the md‐SCD subgroup, whereas this correlation was absent in the sd‐SCD subgroup, further suggesting a higher risk for the md‐SCD subgroup and potential Aβ pathology mechanism. These might indicate that in the NC and sd‐SCD phases, glucose metabolism remains relatively high with limited pathological deposition, whereas in md‐SCD phase, pathological deposition and metabolic alterations become more pronounced. Moreover, among all individuals within the md‐SCD group, reduced glucose metabolism in the ACG.R was associated with decreased AVLT‐N7 scores while these correlations were not present in sd‐SCD group. The same correlation with AVLT‐N7 scores was seen in the MTG.R across all SCD individuals. The mechanism on metabolic differences between sd‐SCD and md‐SCD and relevance with plasma and scales need further exploration. Reduced FDG‐PET brain metabolism was more pronounced in individuals with positive Aβ and tau markers than in patients without reduced cognitive performance [62]. Similar findings were seen in aMCIs that an AD high‐risk FDG‐PET pattern exhibited a lower total plasma Aβ42/40 ratio [63]. Plasma Aβ42/40 has demonstrated value in detecting brain Aβ pathological changes, indicating that Aβ might be the mechanism for our diverse correction results between sd‐SCD and md‐SCD [64, 65, 66]. The results were consistent with research explored by others who reported that plasma Aβ42/40 and FDG‐PET biomarkers were consistently negatively correlated with Aβ‐PET status, and low plasma Aβ42/40, which showed more obvious cognitive decline, increasing the risk of progression to dementia, elucidating potential Aβ pathological mechanisms [63, 67]. Concurrently, in advance of abnormalities in CSF Aβ42/40 and Aβ‐PET, plasma Aβ42/40 abnormalities might emerge, suggesting a potential ability for earlier detection of Aβ [68]. To be more specific, amyloid‐β, an upstream factor in AD pathogenesis, accumulates more in md‐SCD individuals compared to sd‐SCD, potentially elucidating the pathological basis for metabolic differences in SCD between the two groups [8, 69, 70]. Given the above, we thought that the differences in glucose metabolism between sd‐SCD and md‐SCD along with the potential as a new biomarker for predicting conversion are reliable, which might herald disease progression and cognitive decline. Further research is encouraged to clarify the involved mechanisms underlying the difference between the sd‐SCD and md‐SCD.

The current study has several limitations. First, due to its single‐center design, the sample size was relatively small, which restricted our statistical analysis. Consequently, it is imperative to validate these findings in a larger sample in future investigations. Second, the FDG‐PET data with longitudinal follow‐up were not obtained in this study. Third, considering the cross‐cultural effects across different ethnicities, the western cohorts will be involved in the future. Furthermore, the vascular risk factors would be applied to investigate the potential risk of AD continuum cognitive function variations. Follow‐up data from the scale used in this study also validated the biomarker of the SCD subtype to some extent, with limitations but little impact. Moving forward, we will further expand the sample size and obtain the FDG‐PET imaging data with follow‐up for further longitudinal analysis, which outlines our prospective direction. Despite these limitations, the results of the differential studies we explored further stratify SCD and facilitate the identification of SCD at greater transform risk, with a view to complementing the ATN framework for early clinical recognition of AD, personalization, and precision medicine for AD.

## 5 Conclusion

The present study explored differences in glucose metabolism between sd‐SCD and md‐SCD individuals, with ITG.R, MTG.R, ACG.L, and ACG.R progressively decreasing along the cognitive continuum. The SUVR of ACG.R correlated with plasma Aβ42/40, while MTG.R and ACG.R correlated with the degree of recognition of memory functions. In addition, SCD participants who were in the md‐SCD group or positive for glucose metabolism in four regions, plasma Aβ42/40, and p‐tau181 exhibited an increased risk of conversion. Based on our findings, which stratify SCD more finely, md‐SCD or SCD with positive biomarkers might represent a later period, high‐risk phase of SCD with a higher risk of cognitive decline. This refined stratification difference of SCD, complementing the ATN framework, provides a new valuable clinical biomarker for timely diagnosis, identifying high‐risk individuals, and predicting cognitive conversion.

## Author Contributions

Min Wei, Luyao Wang, Xianfeng Yu, and Wenjing Hu designed the study, collected, organized, and analyzed the data, reviewed literature, and drafted manuscript. Ying Han and Jiehui Jiang devised the study, managed data, revised, and reviewed the manuscript. Min Wang, Qi Zhang, Tengfei Guo, Jiayi Zhong, and Chenyang Li organized the data and revised the manuscript. All authors read and approved the final manuscript.

## Ethics Statement

The study was approved by the Medical Ethics Committee of Xuanwu Hospital, Capital Medical University, and was conducted in accordance with the Helsinki Declaration.

## Conflicts of Interest

The authors declare no conflicts of interest.

## Supporting information


Data S1.

## Data Availability

The data used to support the findings of this study are available from the corresponding author upon reasonable request.
