# Supplementary material for: Differences in Glucose Metabolism Between Single Memory Domain and Multidomain Subjective Cognitive Decline: A Longitudinal Study From SILCODE
Source: CNS Neurosci Ther. 2025 May 27;31(5):e70264. doi: 10.1111/cns.70264 (PMC12116337; doi:10.1111/cns.70264)
Supplement: Supplementary file 1 — Data S1. [file CNS-31-e70264-s001.docx]

**Supplementary Materials**

**Table S1** General characteristics of the SCD participants in the follow-up.

|  |  | Follow-up | |  | *p* value |
| --- | --- | --- | --- | --- | --- |
|  |  | sd-SCD | md-SCD |  | sd-SCD vs md-SCD |
| Sex (M, n%) |  | 11 (31.43%) | 13 (33.33%) |  | 0.861 ^a^ |
| Age (year) |  | 69.171 ± 4.586 | 70.795 ± 5.312 |  | 0.218 ^c^ |
| Education |  | 13.229 ± 2.636 | 12.795 ± 2.858 |  | 0.499 ^c^ |
| APOEε4 (carrier, n, %) |  | 7 (20.00%) | 16 (41.03%) |  | 0.051 ^a^ |
| Memory ^d^ (Yes; n, %) |  | / | / |  | / |
| Language ^d^ (Yes; n, %) |  | / | / |  | / |
| Plan ^d^ (Yes; n, %) |  | / | / |  | / |
| Attention ^d^ (Yes; n, %) |  | / | / |  | / |
| Others ^d^ (Yes; n, %) |  | / | / |  | / |
| Plasma Aβ42/40 |  | 0.061 ± 0.011 | 0.054 ± 0.012 |  | 0.019 ^b^ |
| Plasma p-tau181 |  | 2.035 ± 0.738 | 2.913 ± 1.733 |  | 0.065 ^c^ |
| Plasma NfL |  | 15.875 ± 5.486 | 22.031 ± 12.071 |  | 0.053 ^c^ |
| Plasma GFAP |  | 112.653 ± 40.735 | 133.789 ± 70.707 |  | 0.513 ^c^ |
| AVLT-N5 |  | 7.970 ± 2.114 | 6.889 ± 2.605 |  | 0.064 ^b^ |
| AVLT-N7 |  | 22.485 ± 1.698 | 21.500 ± 2.720 |  | 0.087 ^c^ |
| VFT |  | 19.848 ± 3.144 | 17.722 ± 3.874 |  | 0.015 ^b^ |
| BNT |  | 26.394 ± 1.619 | 25.722 ± 2.480 |  | 0.248 ^c^ |
| STT-A |  | 55.636 ± 21.604 | 69.333 ± 24.422 |  | 0.004 ^c^ |
| STT-B |  | 132.303 ± 41.315 | 170.139 ± 73.138 |  | 0.005 ^c^ |
| MMSE |  | 28.719 ± 1.250 | 27.576 ± 2.319 |  | 0.039 ^c^ |
| HAMA |  | 4.939 ± 4.023 | 7.756 ± 7.237 |  | 0.153 ^c^ |
| HAMD |  | 3.455 ± 4.258 | 5.111 ± 4.566 |  | 0.067 ^c^ |
| GDS |  | 2.576 ± 2.278 | 3.833 ± 3.325 |  | 0.103 ^c^ |
| Plasma Aβ42/40  (positive, n, %) |  | 13 (43.33%) | 22 (73.33%) |  | 0.018 ^a^ |
| Plasma p-tau181  (positive, n, %) |  | 8 (30.77%) | 13 (44.83%) |  | 0.284 ^a^ |

Note: ^a^Chi-square test, ^b^*t*-test, ^c^Mann-Whitney *U* test, ^d^SCD-I domains (decline in memory, language, plan, attention, and others); *p* value: significant differences.

The Subjective Cognitive Decline Interview (SCD-I), which assess the five different cognitive domains (memory, language, plan, attention, and others) of SCD subjects. All interviews were administered by trained research physicians. The interviewer asked for specific changes of mental abilities during the past few years. Firstly, memory: “Has your memory become worse?”; Secondly, language: “Do you have increasing word finding difficulties?”; Thirdly, plan: “Do you have increasing difficulties planning ahead or staying organized?”; Next, attention: “Do you make increasingly more errors when not paying full attention to a task?”; Next, others: “Have you noticed any other cognitive changes? Please describe briefly.” For each endorsed question, the physicians also asked for the details such as onset time, concerns, comparison with peers, and the history of visiting physician due to these changes.

**Abbreviations**

SCD, subjective cognitive decline; sd-SCD, single memory domain SCD; md-SCD, multidomain SCD; NC, normal control; CI, cognitively impaired; MCI, mild cognitive impairment; AD, Alzheimer’s disease; NIA-AA, National Institute on Aging-Alzheimer’s Association; SILCODE, Sino Longitudinal Study on Cognitive Decline; MRI, magnetic resonance imaging; APOE, Apolipoprotein E; FOV, field of view; TR, repetition time; TE, echo time; MNI, Montreal Neurological Institute; SUVR, standardized uptake value ratio; AAL, Anatomical Automatic Labeling; GRF, Gaussian random field; HAMA, Hamilton Anxiety Rating Scale; HAMD, Hamilton Depression Rating Scale; GDS, Geriatric Depression Scale.
